# A Q-method approach to perceptions of professional reasoning in occupational therapy undergraduates

**DOI:** 10.1186/s12909-021-02710-y

**Published:** 2021-05-07

**Authors:** Luis-Javier Márquez-Álvarez, José-Ignacio Calvo-Arenillas, Estíbaliz Jiménez-Arberas, Miguel-Ángel Talavera-Valverde, Ana-Isabel Souto-Gómez, Pedro Moruno-Miralles

**Affiliations:** 1 Faculty Padre Ossó, c/Prao Picón S/N, 33008 Oviedo, Spain; 2 Department of Nursing and Physiotherapy, Universidad de Salamanca, Escuela Universitaria de Enfermería y Fisioterapia, 37007 Salamanca, Spain; 3 Health Integration and Promotion Research Unit (INTEGRA SAÚDE), Faculty of Health Sciences, University of A Coruña, 15006 A Coruña, Spain; 4 University School of Social Work, Universidade Santiago de Compostela, Santiago de Compostela, Spain; 5 Department of Nursing, Physiotherapy and Occupational Therapy, University of Castilla-La Mancha, Talavera de la Reina, Toledo, Spain

**Keywords:** Professional reasoning, Q-method, Occupational therapy / education, Clinical decision-making, Problem solving, Students, health occupations

## Abstract

**Background:**

Professional reasoning provides a firm basis for the development of teaching and assessment strategies to support the acquisition of skills by healthcare students. Nevertheless, occupational therapy educators should use diverse methods of learning assessment to examine student learning outcomes more fully with an evaluation that supports the overall complexity of the process, particularly learners’ subjective experience. The aim of this article is to identify the range of perspectives among occupational therapy undergraduates regarding terms or concepts that are key for improving their professional reasoning.

**Methods:**

Q-methodology was used to address the aim of the study. A concourse relating to a series of ideas, phrases, terminology, and concepts associated with various studies on professional reasoning in occupational therapy, specifically on students in this field, was generated. The terms that had the clearest evidence, the most relevance or the greatest number of citations in the literature were collected (*n* = 37). The P-set was assembled by non-probabilistic sampling for convenience. It comprised undergraduate university students in occupational therapy. Factor analysis was conducted using Ken-Q Analysis v.1.0.6, reducing the number of Q-sets to smaller groups of factors representing a common perspective.

**Results:**

Through statistical analysis of the Q-sorts of 37 occupational therapy students, 8 default factors were identified. The four factors in accordance with the selection criteria were rotated by varimax rotation to identify variables that could be grouped together. Each viewpoint was interpreted, discussed and liked to different aspects of professional reasoning in occupational therapy.

**Conclusions:**

The observed perceptions were linked to the various aspects of professional reasoning that have been widely discussed in the occupational therapy literature. For most of the students, there was a strong correspondence between the narrative, interactive and conditional aspects of the various components.

## Background

In occupational therapy, professional reasoning is defined as the process by which professionals plan, direct, carry out and reflect on the client’s treatment [1, 2]. Its importance is based on its relationship with professional practice, which gives the professional the ability to manage the process of assessment, planning and implementation of the intervention, structuring it within its environment and influencing the effectiveness of the work performed [1, 3, 4].

The development of effective professional reasoning has been the primary objective of occupational therapy studies for many years [5, 6], and clinical courses provide the opportunity to refine these skills [6–8].

As Márquez-Álvarez et al. [9] explain, one of the main areas of research in the literature over the years has been the study of these professional reasoning skills in students, amounting to 20% of publications on professional reasoning in occupational therapy. Nevertheless, few references discuss this topic from the subjective perspective of the student. Neistadt [10] was the first to discuss this topic, starting from the various pedagogical tools that improve the acquisition of the skills necessary to build expertise in the professional domain [10–13]. Although professional reasoning is not a novel concept, instructors in healthcare tend to focus their teaching on how to think like a professional based on specific terminology and the use of strategies that improve decision-making through reflection. Professional reasoning is a complex and multifaceted concept that is described and used in very different ways by different authors [11, 14]. Clarity regarding its nature and a shared understanding of the range of uses of professional reasoning provide a firm basis for the development of teaching strategies to support the acquisition of this skill by healthcare students [15]. According with Blumberg [16], instructors need to plan how students will practice engaging with content that requires these different types of knowledge and not assume that they will learn the conceptual or procedural knowledge by attending lectures or demonstrations. For this it is necessary to ask students to reflect on their own learning processes and to assess their learning progress.

According to Dutton [17], professional reasoning skills differ significantly between experienced practitioners and new graduates: “The better our understanding of expert practice and how experts reason, the greater our capacity to provide this complex and often tacit knowledge to novices to hasten and progress their journey to expertise” ([4] p14). The importance of professional reasoning in higher education programmes as a mean to develop professional habits, skills and thinking has gained importance over the years as “learning needs to emphasize reflection on thinking rather than just equipping learners with process-following or decision-making skills” ([18] p2). The way in which novices organise their knowledge to analyse and synthesize the information gathered during the initial assessment is an element of primary importance to acquire proper professional reasoning during their academic education [19].

To analyse this reasoning, current theories [20] recognise that both analytical and non-analytical processes work together and interact mutually, so studying reasoning while separating these processes leads to an oversimplification of the conclusions obtained. Instead of attempting to focus on specific issues or attributes like problem solving or decision making, modern learning assessments tend to look for the development of an evaluation that supports the overall complexity of the process [20]. This excludes those studies that are linked to key responses or based on a “single answer” as the best solution to a problem. New concrete approaches – quantitative, qualitative, and mixed – have arisen from this perspective, such as case studies, multiple-choice questions, threshold concepts or other standardised tests [14, 21–24]; however, there is much to explore in the field of occupational therapists’ professional reasoning as many terms and issues are interwoven in the literature [18]. In this terms, Q-sort is one example of a modern assessment that can be used to identify and potentially assess learner knowledge about professional reasoning. There seems to be a need to identify the limits and edges of professional reasoning and its evolution over the years as many of its determinants remain unknown and lack consensus [18, 25–27]. To educate quality practicians, there is a need to adequately explore the insights of undergraduate occupational therapy students to ensure the efficiency of educational programmes and to minimize the gap between study and professional/clinical practice [18, 21]. From this point of view, Q-sort is a method of assessment that aligns with measuring learning outcomes in regard to development of professional reasoning.

The aim of this article is to identify the range of perspectives among occupational therapy undergraduates regarding terms or concepts that are key for improving their professional reasoning. How do undergraduate occupational therapy students perceive the relevance of the different elements that interact within their professional reasoning learning?

To answer these questions, we chose to use Q-methodology, which incorporates aspects of both quantitative and qualitative techniques to examine human subjectivity [28]. The complementary treatment of quantitative and qualitative methods provides subjectivity within a rigorous and objective process [29].

Q-methodology is a tool that enables a better understanding of people’s perspectives and their beliefs, which are generated and explored through a specific method of data collection and statistical analysis [30]. It has been applied to the improvement of higher education programmes and to quality and healthcare policies with good results [31, 32]. Its use in educational settings allows the experience and perspective of students to be explored as a means of improvement and allows variations to be examined with scientific evidence. Therefore, it is possible to identify participants’ viewpoints by requesting individuals to undertake an operant procedure (sorting related statements).

## Methods

### Design

Q-methodology enables the subjectivity of participants to be preserved through an objective process in which each participant provides his or her perspective by ordering different terms or phrases according to a predetermined study question [33]. It uses a specific statistical method called factor analysis, that reduces a large number of variables into a smaller number of factors to group people according to how they interpret statements about a topic [31]. From this position, the current study is descriptive, exploratory, and transversal, centred on individual and subjective perspectives.

### Concourse development

A concourse relating to a series of ideas, phrases, terminology, and concepts associated with various studies on professional reasoning in occupational therapy, specifically on students in this field, was generated. This review was conducted retrospectively according to the PRISMA methodology [34], and information was collated relating to professional reasoning in occupational therapy students between 1986 and 2020. The formal literature search was conducted across the following databases: OTDBase, CINAHL, Medline, WOS, Embase, Scopus, ISOC, Latindex, LILACS, LivRe, ProQuest, CSIC (Spanish National Research Council) and Dialnet. An initial search strategy (October 2020) was created for Medline (using PubMed) and was adapted to each search: (“Occupational Therapy”[Mesh]) OR (“Allied Health Occupations”[Mesh] OR “Allied Health Personnel”[Mesh]) AND (“clinical reasoning” OR “professional reasoning”) AND (students OR undergraduates). After applying the selection criteria, we identified 44 references of interest.

### Development of the Q-set

The Q-set is a set of statements representative of the majority of ideas present in the opinions of the field of study [35]. In this case, to prepare the initial set, there were collected all the terms which were related in any way to professional reasoning according to most cited authors, greatest levels of evidence in the studies and appeared most repeatedly in the specific issues from professional reasoning literature. A group of the study authors (LJMA, MATV and PMM) separately created different sets that were subsequently combined by eliminating duplicates and combining terms with similar definitions or characteristics. Finally, a list of *n* = 37 terms and statements was prepared (Table 1). There was no subsequent modification to this sample.

**Table 1:** Q-set statements and terms prepared for Q-sort

Statement Num.	Statements
1	Cooperative learning
2	Experimental learning
3	Individual learning
4	Self-assessment
5	External aid
6	Functionality of the client
7	Contact with people with disabilities
8	Procedural reasoning
9	Pragmatic reasoning
10	Main diagnosis
11	Disability
12	Illness
13	Ethical reasoning
14	Assessments of the client
15	Professional experiences
16	Academic education
17	Scales and forms to assess
18	Narrative reasoning
19	Interactive reasoning
20	Frames of reference
21	Improve skills as student
22	Mentor
23	Models of practices
24	Aims of intervention
25	Think as therapists
26	Conditional reasoning
27	Images of people with disabilities
28	Intervention planning
29	Clinical practice
30	Main problem of the client
31	Referents in own learning
32	Community reinsertion
33	Problem solving
34	Individual responsibility
35	Roles of the client
36	Routines of the client
37	Specific vocabulary and terminology

### Selection of the P-set

The P-set (set of participants) [36] was assembled by non-probabilistic sampling for convenience. It comprised undergraduate university students in occupational therapy who wished to take part in the study. The students had some knowledge of the discipline and of the terms offered in the list (at least second year of study) and had access to computer equipment to complete the data collection forms.

Given that the Q-methodology seeks to identify the different opinions within this group of participants, there is no need to make a maximum sample size calculation. In accordance with the literature and following the indications of Watts & Stenner [37], an approximate 1:1 ratio of terms to people interviewed is required to conduct the study. The number of participants should not exceed the number of terms or phrases used. Therefore, 37 students participated in the study, including any volunteer who was willing to take part in the study was included until reach the specified number.

### Q-sort

The participants were recruited in November 2020 from the different courses in the Degree in Occupational Therapy from Faculty Padre Ossó in Oviedo. They assessed the Q-set statements on a continuous scale with a quasi-normal distribution between “most relevant” and “least relevant”. A preliminary session was used to explain all possible doubts and to discuss the meaning of all the statements, although all participants had read them previously. The following premise was presented to them to perform the sort:

*“The aim of this activity is to know your own perceptions about the importance of some terms in using clinical reasoning in occupational therapy. Following this text, you are going to see a table that represents a normal curve (like a Gaussian distribution), and each column has its own value (for example, F has a value of 1). (*

**Fig. 1:**
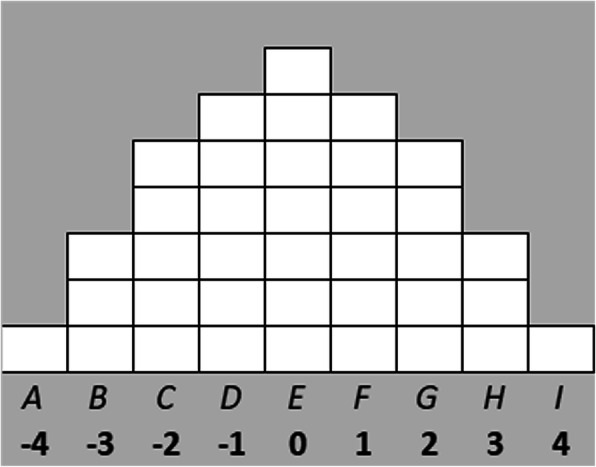
Distribution scale for the statements, in accordance with the instructions to participants

Figure 1*)*

*Next, you will find a list of 37 terms. I would like you to put each of them in the square where, according to your preference, it should be placed in terms of relevance for clinical reasoning in occupational therapy. Remember, you cannot repeat any of the squares or use more than the given ones (you can use the numbers to guide you). For example, if you put the professional experience term over the "I" column, it means that it is very important to you at the time for your own reasoning. However, maybe you think it is not so important, so you could put it in the "E" column, or maybe you think it is not important at all, so you should put it in the "A" column”.*

The 37 statements were classed on a scale with a [− 4, + 4] distribution of ranks, with 9 columns in total (Fig. 1).

The statements were classed on the scale using the Microsoft Excel 365 program given that all participants had access to this software. Statements should normally be classed using cards, but the method using Microsoft Excel was chosen for reasons of hygiene due to the COVID-19 situation. At the end of each document, the participants had a space in which they could include comments about statements they believed should have been included and that could be relevant as well as any observations regarding how they had made their choice.

### Factor analysis

The factor analysis was conducted using Ken-Q Analysis v.1.0.6 (available from https://shawnbanasick.github.io/ken-q-analysis/). Factor analysis was used to reduce the number of Q-sets to smaller groups of factors representing a common perspective [36]. The process consisted of the following selection criteria for the extraction of factors based on the criteria of Garbellini et al. [33] and their review of the work of Chee, Lee, Patomella & Falkmer [37] and Thompson, Elliot, Willis, Ward, Falkmer et al. [38]. An additional step (no. 5) was added as a possibility given the characteristics of the software.

**Fig. 2:**
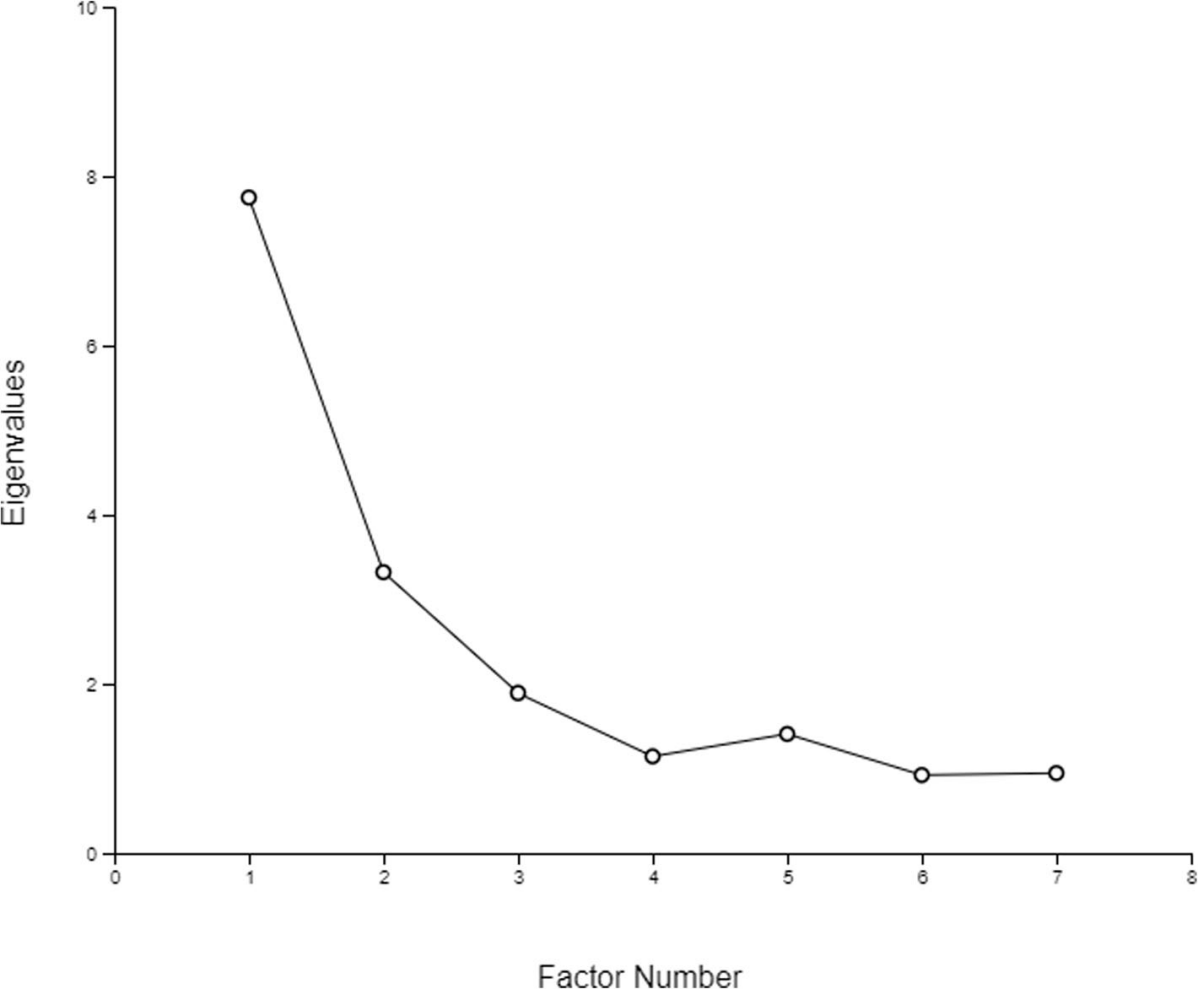
Eigenvalues of the retained factors extracted from the software

1. The starting point was the default number of factors extracted by the Ken-Q Analysis software, a total of 7 factors.
2. Factors with an eigenvalue greater than 1.0 were included.
3. At least two significant factor loadings were required for each retained factor.
4. The cross-product of the two highest loadings should be greater than twice the standard error (SE) (Humphrey’s rule). SE was calculated using the formula SE = 1/√*n*, where *n* = number of statements in the Q-set. Therefore, loadings of 2 × 1/√37 (factor loadings > 0.3288) identified Q-sorts correlated with each factor.
5. In the auto-flagging of factors, all with *p* > 0.01 were excluded.
6. All factors displayed prior to the levelling of the scree plot (Fig. 2) should be retained.

### Interpretation of factors

The interpretation of each factor was performed in three steps: a) each factor was analysed at a general level; b) the statements at the two extremes (values of − 4, − 3, + 3 & + 4) were analysed together to observe the counterpoint of perceptions and compare them to the existing literature.

This comparison enabled the identification of the various viewpoints and their influence on the student’s learning as well as how the result could be usefully extrapolated.

## Results

Responses were obtained from 15 s-year students and 22 third-year students, 8 males and 29 females, with an age of *x̄* = 19.757 (sd = 1.116). The second-year students had just started to examine clinical cases oriented towards specific areas of clinical practice (older patients, joint disease and mental health), while the third-year students had one more year of experience.

**Fig. 3:**
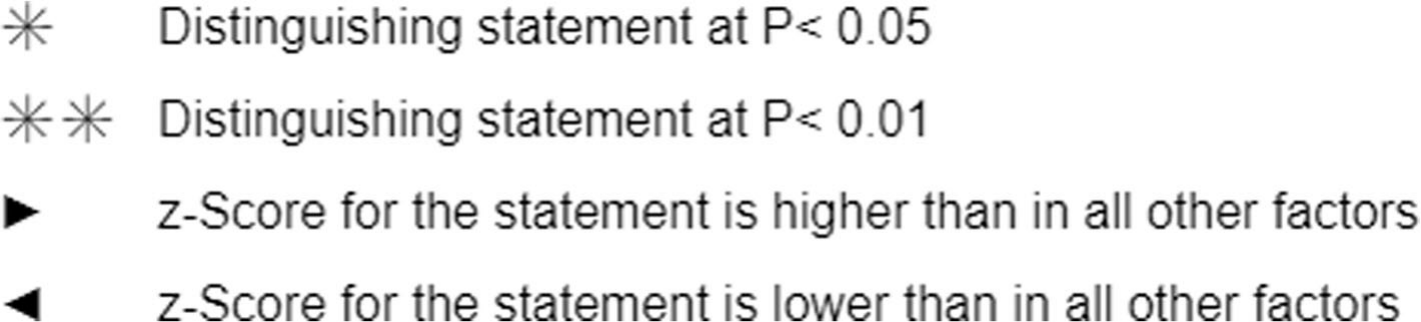
Legend for the viewpoints

The analysis of the Q-sorts of the 37 participants created 8 default factors that were centred on 4 viewpoints in accordance with the selection criteria (see Table 2 and Fig. 2). All viewpoints included Q-sorts from both years, showing representability of the student body in general; however, it was not possible to show an effect of additional practice or study. The four selected factors were rotated by varimax rotation to identify variables that could be grouped together and to maximize the set of mutually distinct observations [39] (see Fig. 3 for the legend of viewpoints).

**Table 2:** Application of selection criteria following Garbellini et al. [28]; Y = Yes; N=No

Selection criteria							
*Default factors*	**1**	**2**	**3**	**4**	**5**	**6**	**7**
*Eigenvalue (value needed > 1.0)*	7.746	3.321	1.893	1.146	1.41	0.924	0.948
*Number of factor loading > 0.3288*	11	2	5	1	3		
*Humphrey’s rule succeeded*	Y	Y	Y	N	Y	–	–

Following the steps in the method, each factor will be summarized by the statements at the two extremes (values of − 4, − 3, + 3 & + 4),

### Retained factor 1: viewpoint 1 – focus on client and results

**Fig. 4:**
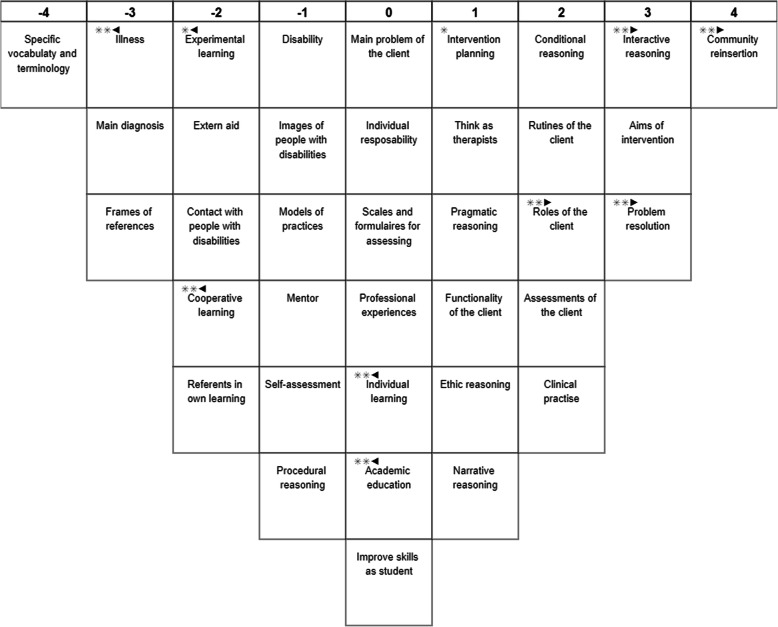
Viewpoint 1 matrix extracted by the Ken-Q Analysis software

The first viewpoint (Fig. 4) was named because of the counterpoint of two aspects that are very different for training but have statements that are closely linked to the client and to the client’s experience in clinical and day-to-day terms.

On the one hand, aspects of client contact are found at the high end of the curve, such as “*interactive reasoning*”, “*community reinsertion*” and “*roles of the client*”. At the other end of the scale are statements such as “*frames of reference*”, “*main diagnosis*” and the concept of *“illness”*. As shown in the figure, in many cases, the priority for resolution of the clinical case does not seem to be understood as involving learning but as a problem to be solved.

### Retained factor 2: viewpoint 2 – focus on academic learning

**Fig. 5:**
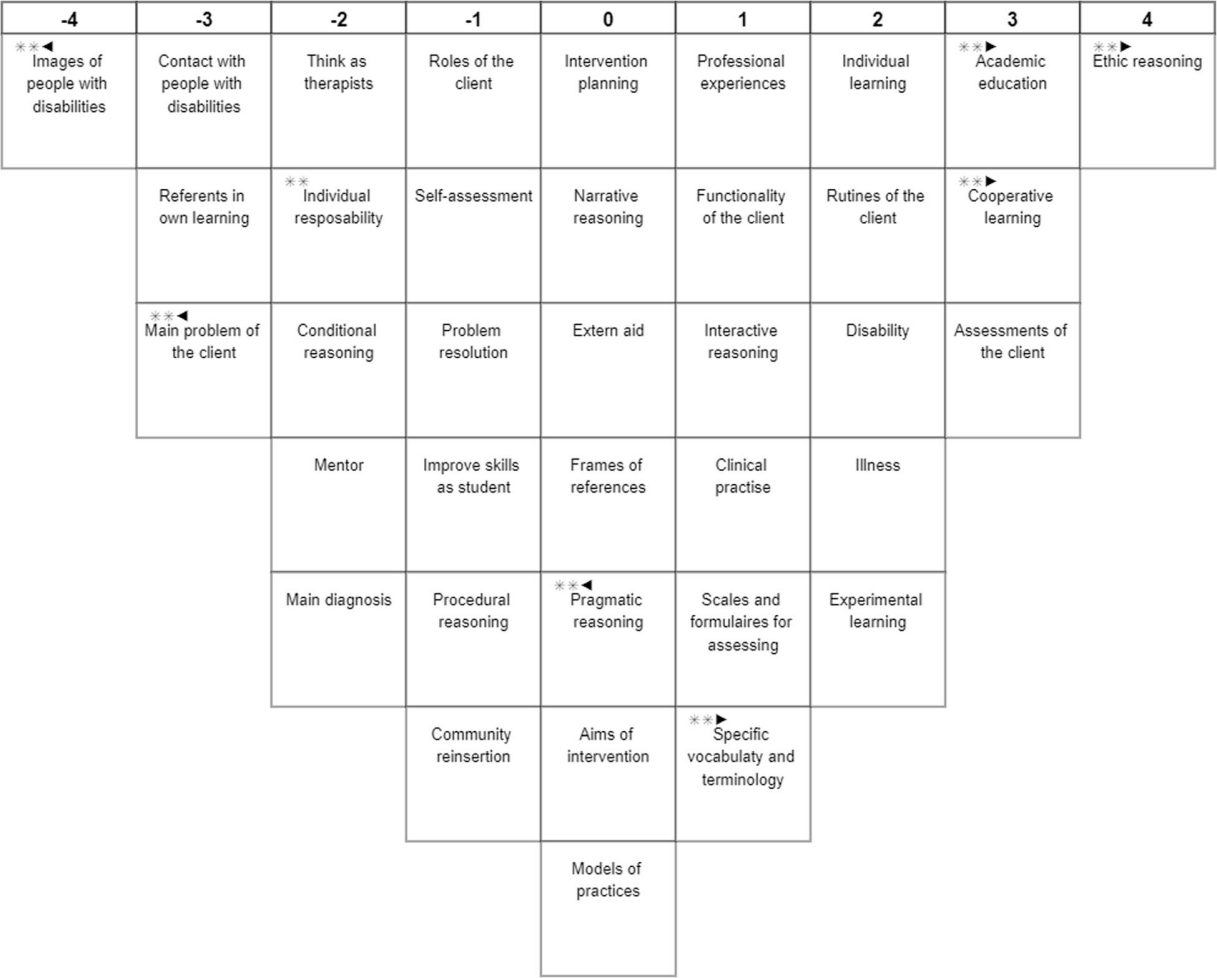
Viewpoint 2 matrix extracted by the Ken-Q Analysis software

In contrast to the first viewpoint, there appears to be a greater balance of academic and day-to-day aspects in this view (Fig. 5). It is the only one in which “*specific vocabulary and terminology*” receives some importance, but only in the centre of the bell curve and without extreme values.

The statements viewed as most important are “*ethical reasoning*”, “*cooperative learning*” and “*academic education*”. Statements related to personal care, such as “*images of people with disabilities*” or “*contact with people with disabilities*”, are seen as less important. There seems to be a more academic view in which cases are resolved in a methodical way linked to the learning process.

### Retained factor 3: viewpoint 3 – focus on the process

**Fig. 6:**
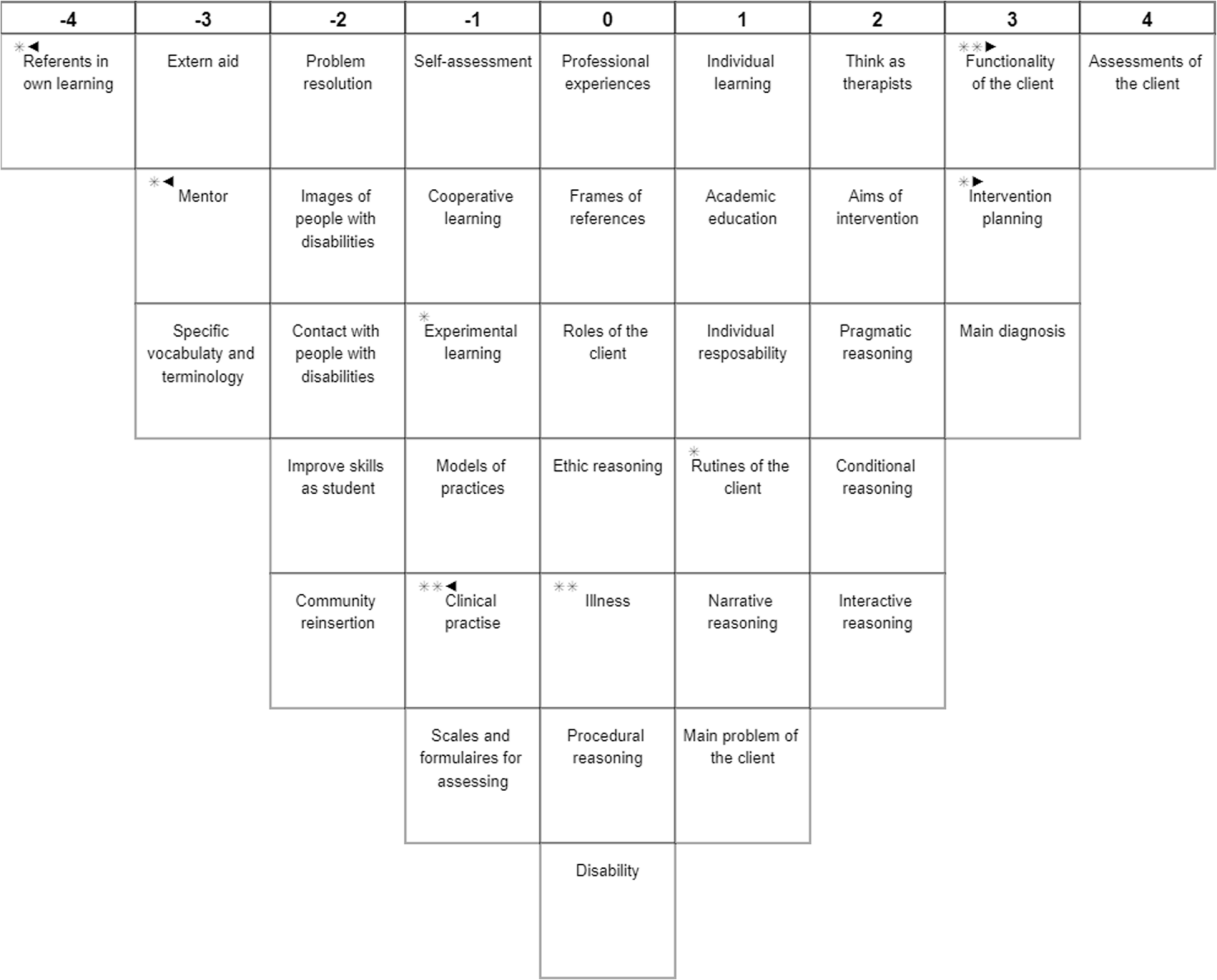
Viewpoint 3 matrix extracted by the Ken-Q Analysis software

The title of this viewpoint (Fig. 6) comes from the large number of components linked to the occupational therapy process in general, which is perceived as more important. Aspects such as “*aims of intervention*” and “*think as therapists*” are seen as relevant, but not as much as the process of assessment and intervention, with the principal axes in “*intervention planning*” and “*functionality of the client*”. On the other hand, statements such as “*referents in own learning*” and “*mentor*” are rated negatively.

### Retained factor 5: viewpoint 4 – focus on nonspecific learning

**Fig. 7:**
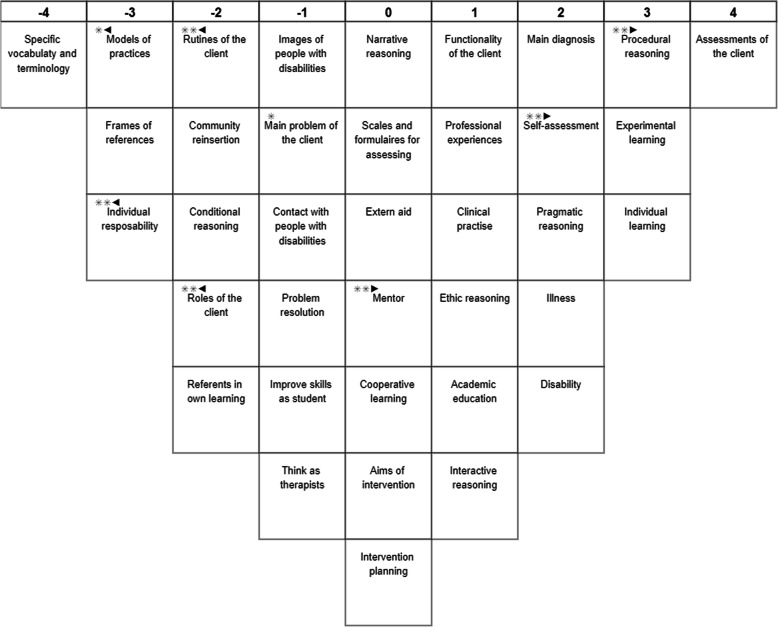
Viewpoint 4 matrix extracted by the Ken-Q Analysis software

In viewpoint 4 (Fig. 7), there is a clear vision of the mechanistic aspect governing reasoning. The most highly rated aspects are “*procedural reasoning*” and “*assessments of the client*”, with “*main diagnosis*” and “*illness*” completely opposed to the other viewpoints.

Constructs that are entirely relevant for learning professional reasoning, such as “*models of practice*” and “*frames of reference”*, as well as individual aspects of the client’s life, such as “*roles*” or “*routines*”, are found in the lowest-rated areas.

## Discussion

To our knowledge, this is the first study to explore professional reasoning in undergraduate students from a Q-methodology perspective. Q-methodology allows us to explore subjectivity from students’ point of view and to better understand their areas of priority to improve their training [40, 41]. Its use enables the exploration of beliefs about the user, the process, and users’ own training and reflection on the aspects that they consider necessary. This approach is in accordance with the purpose of our research question because it develops not only the complexity of professional reasoning but also the possible ranks or comparisons in terms of unanimous statements. Professional reasoning is a key aspect of training within academic programmes that prepares students to take on professional responsibilities. There is a need to develop such programmes to improve the development of students’ abilities, which is vital for professional life [42, 43], and to use innovative assessments of student learning, such as Q-sort, to understand learners’ perspectives and experiences.

Referring to our research question about how students perceive the relevance of the different elements that interact within their professional reasoning learning, it seems there is no consensus even for the same programme of study. Programmes should emphasise different priorities to develop professional reasoning to the extent of a quality practician’s abilities, but the personal context of each student can be seen as biased in that education.

This can be understood with reference to Neistadt’s analysis [11] of the methodological requirements for the teaching of skills related to professional reasoning, which include the use and development of specific terminology to generate precise thought processes early on and to provide a certain capacity for self-evaluation: “An important reason for building a language to describe the often tacit thought processes of occupational therapists is so that expert therapists can communicate more easily with students and novices and thus promote reasoning skills among students” ([4] p6). Nevertheless, in three of the four viewpoints, the statement *“specific vocabulary and terminology”* was placed in a low position. This could be because training programmes are not delivered appropriately or because their impact on students is lower than it should be.

On the other hand, the overall visions of each of the viewpoints take on meaning and comparative capacity when examining concepts related to aspects of reasoning in occupational therapists. An “aspect” refers to the different perspectives on both the nature of the process and the focus or content about which therapists reason. The aspects or tracks of reasoning in occupational therapy include procedural, interactive, conditional, narrative, pragmatic, scientific, ethical, and diagnostic reasoning [4, 44]. If we aim to explore the perception and relevance of the elements that construct reasoning, the concept of aspects seems to be more relevant than the specific cognitive process because each of the predominant aspects is associated with some key terms. If we compare the different viewpoints using this concept, they all seem to reflect, more or less tacitly, aspects of professional reasoning in the context of occupational therapy students. In Table 3, key concepts of each viewpoint for the discussion are associated with the different aspects of reasoning.

**Table 3:** Key concepts discussed and related to aspects of professional reasoning (VP = viewpoint)

VP	Highly rated		Poorly rated	
	*Statements*	*Aspects of reasoning*	*Statements*	*Aspects of reasoning*
**1**	• interactive reasoning • community reinsertion • roles of the client	Narrative, conditional and interactive reasoning	• frames of reference • main diagnosis • illness	Scientific and procedural reasoning
**2**	• ethical reasoning • cooperative learning • academic education	Ethical and scientific reasoning	• images of people with disabilities • contact with people with disabilities • main problem of the client	Interactive and narrative reasoning
**3**	• assessments of the client • intervention planning • functionality of the client	Pragmatic reasoning	• referents in own learning • mentor	Related to highly pragmatic reasoning
**4**	(Not clearly identified)	–	• specific vocabulary and terminology • models of practise • frame of reference	Procedural reasoning

In this way, viewpoint 1, the majority viewpoint with an eigenvalue of 7.746, prioritises the construction of an occupational life history. A large part of this viewpoint seems to cover the search for autonomy and independence for the person receiving the services, and this search can be seen as more important than the direct learning process. Statements such as “*interactive reasoning*” and “*community reinsertion*”, which are related to the client’s experience, life and future, have their own narrative for students that involve their own life situation. As such, this typology can be seen as a combination of narrative, conditional and interactive reasoning. These aspects promote awareness of the experience of illness and of the various styles of interaction [44].

There is a reflex towards procedural reasoning in the two viewpoints, although in very different ways. Procedural reasoning uses a series of scientific bases on reasoning to create, test and use knowledge to make decisions. Scientific reasoning also partly overlaps with evidence-based practice in that both are concerned with the evaluation and application of research evidence to clinical practice [44]. Depending on whether these bases are specific to occupational therapy or outside its remit, the applicable perspective could be viewpoint 2 or viewpoint 4, respectively. In the former, care is centred on the points of application defined by Schell and Schell as “frame of reference selection”, “occupational profile” and “analysis of occupational performance”; the latter makes reference to “referral” [44]. The former could support learning in occupational therapy in a stronger way, directly using its scientific foundations based on thought aspects that are strongly related to higher cognitive processes and linked to learning. The latter could abstract the student from the direct occupational therapy intervention, with the focus of attention being diagnostic aspects or problems other than occupation.

Finally, in viewpoint 3, the content of a large number of statements seen as important could indicate a more scientific vision related to “procedural reasoning”. Nevertheless, there are statements with a value of + 2 that are related to aspects of direct contact with clinical experience and the client, and almost all statements are related to aspects of higher thought. The negative rating of some statements related to referent figures may suggest the loss of the figure of a teacher or professional that is replaced by a vision of the whole process. This type of viewpoint could be oriented towards self-learning or the need to internalize certain concepts of the occupational therapy procedure more than in the resolution of cases.

Viewpoint 3 is perhaps the more conclusive evidence. It downplays external reference points while searching for individual development and conducting a direct analysis with priorities such as diagnosis, the client’s principal complaint, assessment or functionality. This seems to describe the characteristics of pragmatic reasoning, which reflect an immediate intent to resolve the case [45]. Pragmatic reasoning is centred on the day-to-day realities that occur in the department while considering the contextual factors that inhibit or facilitate therapy [46]. Although it is impossible to see pragmatic reasoning in a simulated exercise because there is no real-life context, it can be inferred from a predisposition to immediate action with components of direct intervention. This reasoning seeks to build awareness among students of aspects of clinical practice; however, there is no framework of ethics or sensitivity beyond that which is purely specific to the occupational therapy process. One of the reasons for this could be students’ early encounter with such pragmatic reasoning.

The appearance of this mode of reasoning tends to be more appropriate towards the end of the course, when there has already been adequate development with respect to basic narrative, interactive and procedural skills [17]. Centring oneself on aspects that are individual or specific could have long-term repercussions, such as a low level of theoretical justification or a lack of regard for significant aspects of the person’s occupational narrative. Because of this, we can conclude that some acquisitional models of professional reasoning skills do not match the previous evidence because last-year students were not included in the study. This situation provides an opportunity to ask whether these personal biases may be related to different cognitive processes, assumptions or temporary event preferences.

It remains necessary to examine these perspectives more deeply, to make new comparisons of viewpoints and to look more closely at each of the components of the various personal variables. Setting each subjective point as an aspect of reasoning to a greater or lesser extent allows us to assess the evolution of a student’s thoughts, to know whether he or she has examined important aspects sufficiently deeply and to assess his or her scientific knowledge and the specificity of interventions in the field of occupational therapy. Finally, designing training programmes based on this evidence could improve the quality of the various professionals in the discipline. Knowledge of students’ perceptions can facilitate the organisation of new educational programmes and subjects, the promotion of different types of reasoning, the prioritisation of clinical aspects vs. theoretical aspects, and the exploration of the differences between an experiential approach and an academic approach. All of this can improve the quality of undergraduates, making their reasoning more proximate to that of practicians and even closing the gap between novices and experts.

### Limitations of the study

This study explored the perspectives and viewpoints on professional reasoning held by occupational therapy undergraduates in Spain. The findings may not be applicable to people with different backgrounds. This is inherent in the aim of a Q-study, which is to explore patterns of subjectivity. The Q-methodology is not intended to develop general knowledge about a population. Our study considers the topic of reasoning from a new dimension to support students’ academic training based on a methodology that has not been explored in this area to date.

## Conclusions

Through the application of Q-methodology, this study identified the perceptions held by the students of the study group concerning the different variables that interact in their training and in the development of professional reasoning in occupational therapy. The perceptions observed could be linked to the various aspects of professional reasoning that have been widely discussed in the occupational therapy literature. There is a strong correspondence between the narrative, interactive and conditional aspects of the various components in a majority of the students. These components reflect the prioritisation of a practice centred on clients’ requirements, their life experience and their future.

It is necessary to examine this topic more deeply with comparisons between courses and study programmes at different universities and other variables that could help create or modify educational policies intended to improve students’ training and to prioritise those constructs that are less highly rated in these perceptions but are still important in practice. It also seems necessary to use innovative assessments of student learning such as Q-sort, to understand learners’ perspectives and experiences. Professional reasoning is key for the development of an intervention plan and a quality practice that is reflected in the care accorded to the client. This understanding supports its acquisition during university studies, enabling the development of professionals who are better qualified for clinical and research practice.

## Data Availability

The datasets used and/or analysed during the current study are available from the corresponding author on reasonable request.
